# Tildrakizumab in the Treatment of Complex and Severe Psoriasis: A Case Series

**DOI:** 10.3390/jcm15166161

**Published:** 2026-08-08

**Authors:** Claudio Marasca, Domenico D’Amico, Claudia Giofrè, Viviana Lora

**Affiliations:** 1 Dermatology Unit, Medical Department, “Antonio Cardarelli” National Hospital, 80131 Naples, Italy; 2 U.O.C. Dermatology, A.O.U. “Renato Dulbecco”, 88100 Catanzaro, Italy; 3 U.O.C. Dermatology, A.O. “Papardo”, 98158 Messina, Italy; 4 Department of Clinical Dermatology, San Gallicano Dermatological Institute (IRCCS), 00144 Rome, Italy

**Keywords:** severe psoriasis, comorbidities, multi-treatment failure, high-impact areas, case series

## Abstract

**Background/Objectives**: Patients with a clinical diagnosis of severe psoriasis complicated by comorbidities such as cardiovascular disease, obesity, metabolic syndrome and/or with the involvement of high-impact areas constitute a clinical challenge. Tildrakizumab is a monoclonal antibody targeting the IL-23/Th17 axis with a proven record of efficacy and safety in these patients. **Methods**: Here we present four cases of patients with severe psoriasis and comorbidities including cardiovascular disease (Case 1), obesity (Cases 2 and 3), metabolic syndrome (Case 3), and/or high-impact areas (Cases 2–4). Three patients (Cases 1–3) had previously received biologics but developed secondary failure or loss of efficacy; one patient was bio-naïve (Case 4). **Results**: The treatment with tildrakizumab in all four patients led to a quick onset (by Week 4 in all cases) of complete and lasting (1 year in Case 1, 4 years in Case 2 and 2 years in Cases 3 and 4) remission with no adverse events reported. **Conclusions**: Tildrakizumab is a valuable therapeutic option for patients with psoriasis and complex clinical situations, and in particular, in cases with obesity and difficult-to-treat lesion locations such as hands, scalp and genitals. Therapeutic success is often linked to an improvement in patients’ quality of life.

## 1. Introduction

Psoriasis is a chronic, immune-mediated inflammatory disease characterised by keratinocyte hyperproliferation and infiltration of inflammatory cells, resulting from complex interactions between genetic predisposition, environmental factors, and immune dysregulation. In addition to cutaneous manifestations, psoriasis is associated with systemic comorbidities such as cardiovascular disease, obesity, metabolic syndrome, psoriatic arthritis, uveitis, non-alcoholic fatty liver disease, chronic inflammatory bowel diseases, anxiety, and depression. Treatment requires long-term strategies to reduce disease burden and improve quality of life [1].

The advances in the understanding of the biology of psoriasis have identified the IL-23/Th17 axis as crucial in the development and maintenance of psoriasis [2], and led to the development of treatments targeting it [3]. Tildrakizumab, an anti-IL-23p19 IgG1 monoclonal antibody, is approved for moderate-to-severe plaque psoriasis with the standard dosing regimen (100 mg at week 0 and 4, followed by subsequent injections every 12 weeks) [4]. A post hoc analysis of the pivotal studies and real-world experience showed increased efficacy of the 200 mg dosage in subgroups of patients with high disease burden or other clinical complexities, particularly in obese or multi-failure patients [5,6,7]. The favourable safety profile and effective, stable long-term clinical response make tildrakizumab a valid therapeutic option, even in patients with comorbidities and involvement of difficult-to-treat areas [5,8,9,10,11,12].

Here, we present a series of clinical cases of patients (Table 1) with severe and difficult-to-treat psoriasis with comorbidities including cardiovascular disease and obesity, in whom treatment with tildrakizumab led to a complete and lasting remission.

## 2. Case Presentation

### 2.1. Case 1—A Patient with Extremely Severe Psoriasis and Comorbidities

A 60-year-old male patient with a significant past medical history presented with extremely severe plaque psoriasis. His comorbidities included dyslipidaemia, systemic hypertension, and a myocardial infarction, which occurred 10 years prior. His psoriasis first manifested 20 years before and had followed a chronic-relapsing course. Initially, the patient was managed with topical corticosteroids and vitamin D derivatives, providing partial symptomatic relief. As the disease progressed, narrowband ultraviolet B (NB-UVB) phototherapy cycles were employed multiple times with minimal lasting benefit. Subsequently, systemic therapy with methotrexate was initiated and continued for approximately 2 years until loss of clinical efficacy and disease control. No joint involvement was reported.

Given the persistence and worsening of the disease, biological treatment was introduced. The patient first received adalimumab, a tumour necrosis factor-alpha (TNF-α) inhibitor, for about 9 months. Although adalimumab initially improved symptoms, the patient ultimately relapsed, prompting a therapeutic switch to secukinumab, an interleukin-17A (IL-17A) inhibitor. The treatment with secukinumab resulted in an initial clinical improvement; however, after approximately 1 year, efficacy diminished, and the therapy was discontinued.

**Figure 1:**
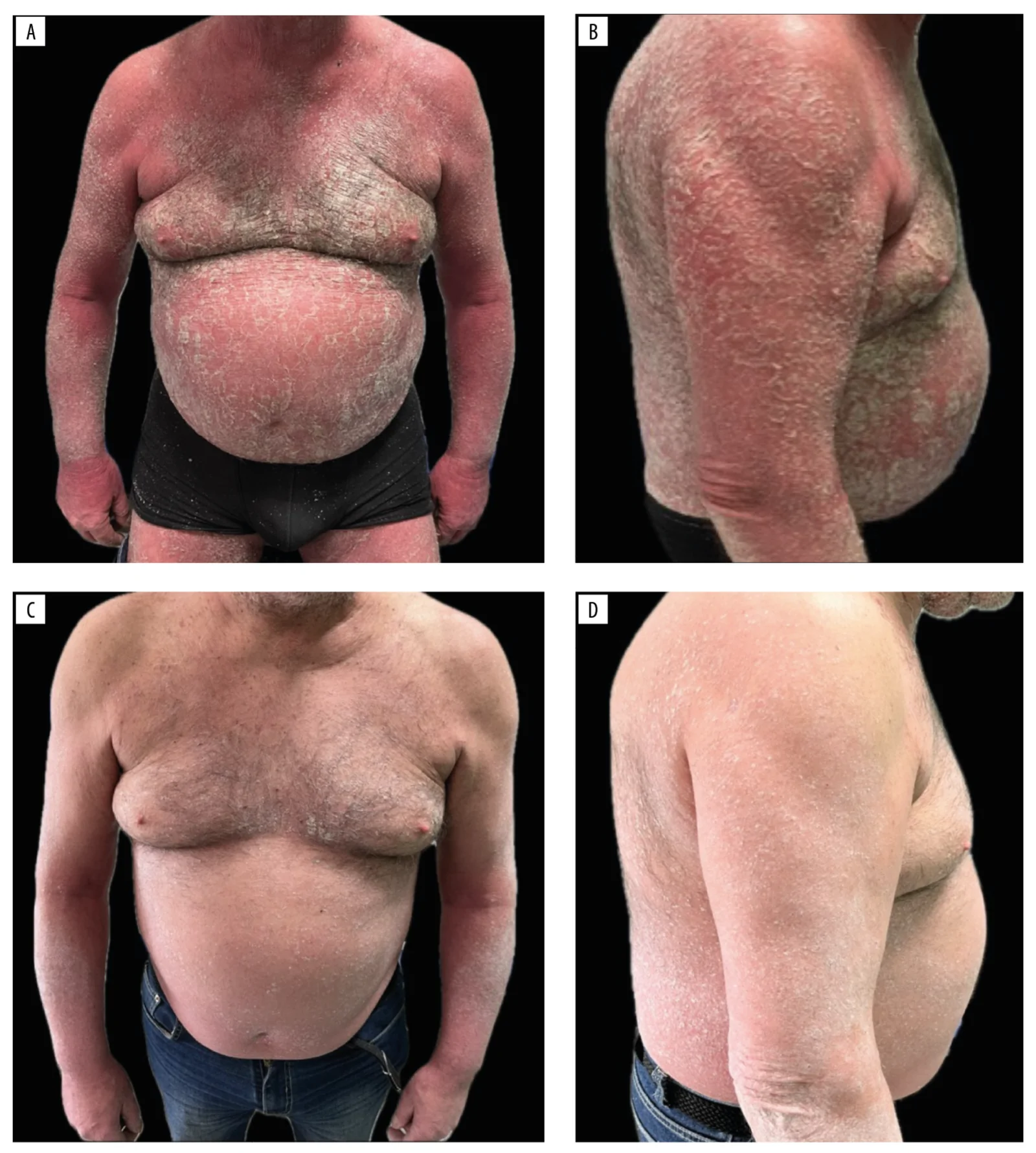
Case 1. (**A**,**B**). Extensive psoriatic lesions at baseline before introducing tildrakizumab therapy. (**C**,**D**). Skin appearance after 6 months of treatment with tildrakizumab.

At this point, the patient exhibited extensive skin involvement, with an estimated 93% of his body surface area (BSA) affected. The clinical severity was rated as extremely high, with a Psoriasis Area and Severity Index (PASI) score of 57 (Figure 1A,B). The treatment plan was revised, and therapy with tildrakizumab 200 mg was initiated. The regimen involved a loading dose of 200 mg administered on Day 1, followed by a second dose after 28 days, and maintenance doses every 84 days thereafter (every 12 weeks). After just 6 months of tildrakizumab administration, the patient demonstrated a near-complete resolution of the psoriatic lesions, with significant reduction in erythema, scaling, and pruritus. The patient reported substantial clinical improvement and enhanced quality of life. No significant adverse events were observed during the treatment course (Figure 1C,D).

### 2.2. Case 2—A Patient with Complex Psoriasis and Metabolic Syndrome

A 46-year-old man, with a maternal and paternal history of psoriasis, was affected by plaque psoriasis without psoriatic arthritis since the age of 16 years. The familial history also included the presence of cardiovascular disease in his father. He was overweight with persistently elevated levels of total cholesterol, triglycerides, and blood pressure.

Over the years, the patient received several biologics, starting with infliximab and subsequently moving to adalimumab, ustekinumab, and secukinumab. Each therapy resulted in an initial clinical improvement, followed by a rapid and irreversible secondary loss of efficacy. As the number of therapeutic switches increased, clinical response diminished, and therapeutic persistence declined. The clinical picture was also complicated by the progressive weight gain (from 90 to 120 kg over 15 years) and the onset of a metabolic syndrome.

**Figure 2:**
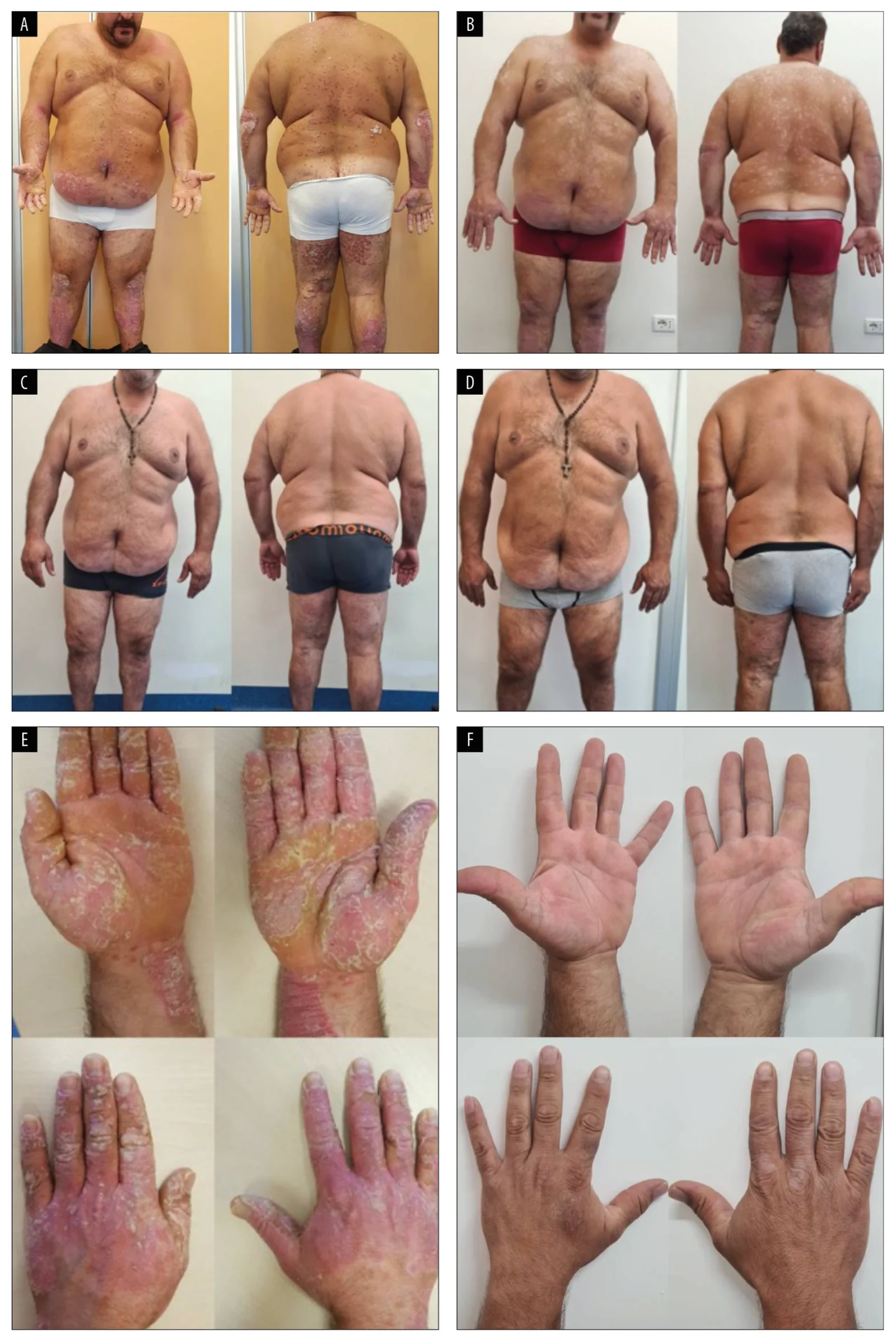
Case 2. (**A**). Week 0: extensive erythematous and desquamative plaques involving the trunk and limbs before treatment with tildrakizumab; (**B**). Week 4: marked reduction in the extent of the plaques and in their erythematous-desquamative component; (**C**). Week 12: complete resolution of the lesions on the trunk and limbs; (**D**). Week 208: continued remission; (**E**). Week 0: severe involvement of hands; (**F**). Week 12: complete resolution of lesions on hands.

In May 2021, while on secukinumab (started in November 2018), the patient experienced a disease flare with extensive erythematous, scaly, and thick plaques on the trunk and limbs (Figure 2A), and a particularly disabling involvement of the hands causing work impairment (Figure 2E). He reported a significant decline in mood due to repeated therapeutic failure. The clinical scores at that time were PASI 24 and Dermatology Life Quality Index (DLQI) 18; the patient weighed 120 kg for a body mass index (BMI) of 38, i.e., had grade II obesity. Laboratory findings included elevated transaminases (GOT: 72 U/L, GPT: 68 U/L), total cholesterol: 260 mg/dL, LDL cholesterol: 140 mg/dL, and triglycerides: 180 mg/dL. Abdominal ultrasound confirmed hepatic steatosis.

Given the disease progression and resistance, a class switch from IL-17 to IL-23 inhibition was considered. Tildrakizumab 200 mg was selected based on available efficacy data suggesting that this higher dosing regimen provides greater benefit in patients weighing ≥90 kg [4]. Therapy was initiated in May 2021, with injections at weeks 0, 4, and every 12 weeks thereafter. A significant clinical improvement was noted by Week 4 (PASI 12, DLQI 6; Figure 2B), and complete disease clearance was achieved by Week 12 (PASI 0 and DLQI 0; Figure 2C). Also, there was an excellent response regarding hands with complete lesion regression (Figure 2F). The patient reported substantial improvement in quality of life and the resolution of depressive symptoms. Body weight remained unchanged; laboratory parameters showed a modest improvement (GOT: 60 U/L, GPT: 56 U/L, total cholesterol: 240 mg/dL, LDL: 130 mg/dL, triglycerides: 160 mg/dL). Treatment continued at 200 mg every 12 weeks. At the most recent follow-up (May 2025), the patient remained in complete remission (PASI 0, DLQI 0; Figure 2D), with stable laboratory results and weight reduction to 110 kg. No adverse events were observed throughout the 4-year follow-up period.

### 2.3. Case 3—Retreatment with Tildrakizumab in a Patient with Severe Psoriasis with Comorbidities

**Figure 3:**
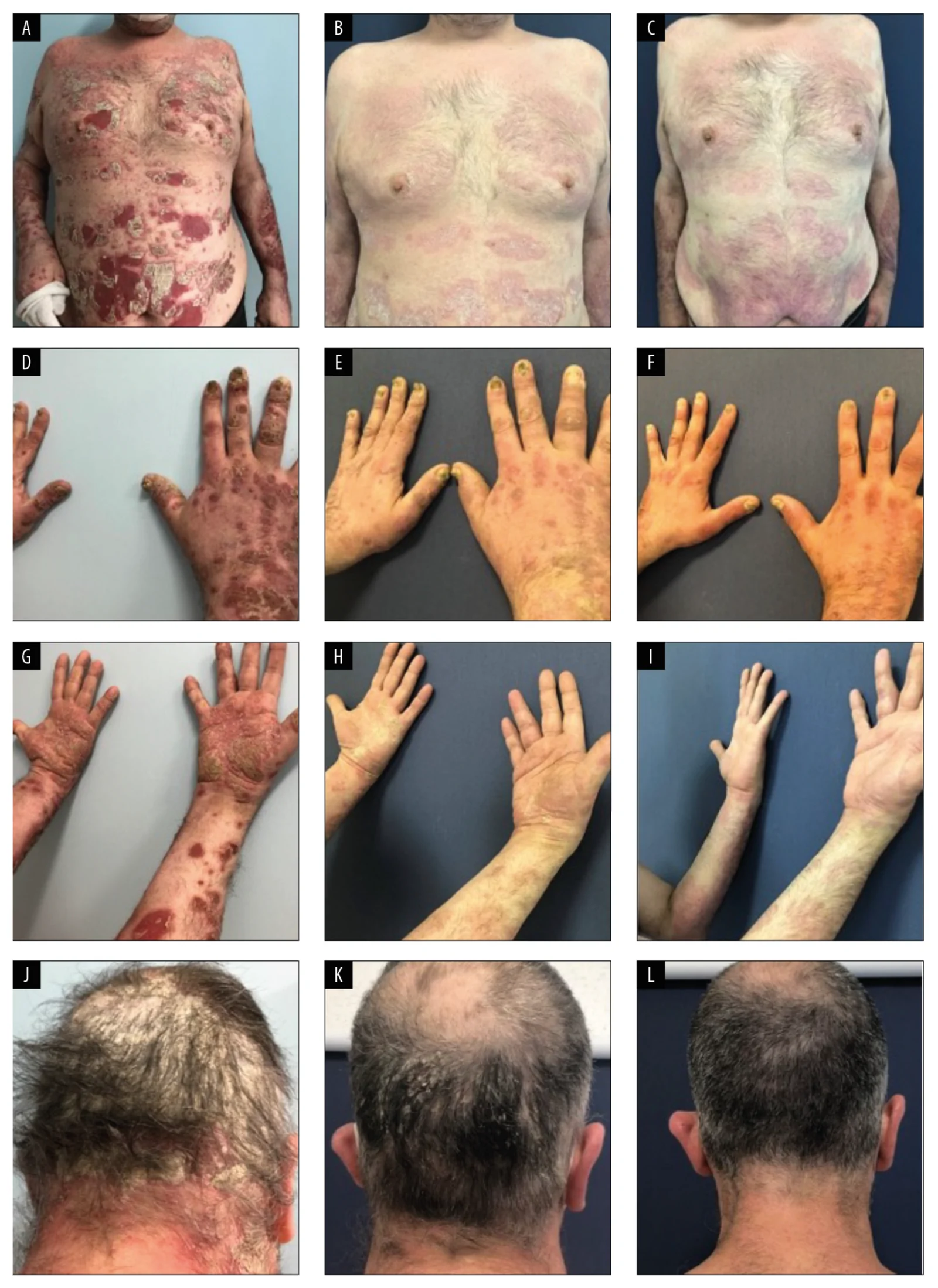
Case 3. (**A**,**D**,**G**,**J**). Week 0: extensive baseline erythematous and desquamative plaques involving the trunk (**A**), hands (**D**,**G**), limbs and head (**J**) before re-treatment with tildrakizumab; (**B**,**E**,**H**,**K**). Week 4: improvement of psoriatic lesions (**C**,**F**,**I**,**L**). Week 108: complete resolution of psoriatic lesions.

This 48-year-old man, with a positive familial history of psoriasis (father and brother), was affected by plaque psoriasis since the age of 28 years. He was a smoker with a past history of hepatitis C (HCV Ab+, HCV RNA−), past drug addiction, uveitis, and depression. Between 2014 and 2021, he was treated with adalimumab, which was discontinued due to secondary inefficacy. In November 2021, the patient was started on tildrakizumab 100 mg and achieved complete remission (PASI 0, DLQI 0); however, he voluntarily discontinued his therapy with tildrakizumab after 1 year of treatment. The treatment interruption lasted 12 months and led to the worsening of his psoriasis (PASI 57.6, DLQI 30, BSA 80% and BMI 33.95; Figure 3A,D,G,J) with the involvement of hands and scalp (baseline Psoriasis Scalp Severity Index [PSSI] 70). Tildrakizumab 200 mg was therefore restarted. At Week 4 of re-treatment, there was an important improvement in psoriatic lesions (PASI 11.5; DLQI 0; BSA 5%; PSSI 30; Figure 3B,E,H,K). By Week 108, the resolution of the psoriatic lesions was complete (PASI 0; DLQI 0; PSSI 0; BSA 0%, Figure 3C,F,I,L). The patient regained confidence and motivation, and remained adherent to the treatment.

### 2.4. Case 4—A Bio-Naïve Patient with Severe Plaque Psoriasis and Intense Itch

A 49-year-old male patient with a history of psoriasis since the age of 23 had exclusively received topical therapies and a limited course of acitretin, which was discontinued due to severe ocular dryness.

**Figure 4:**
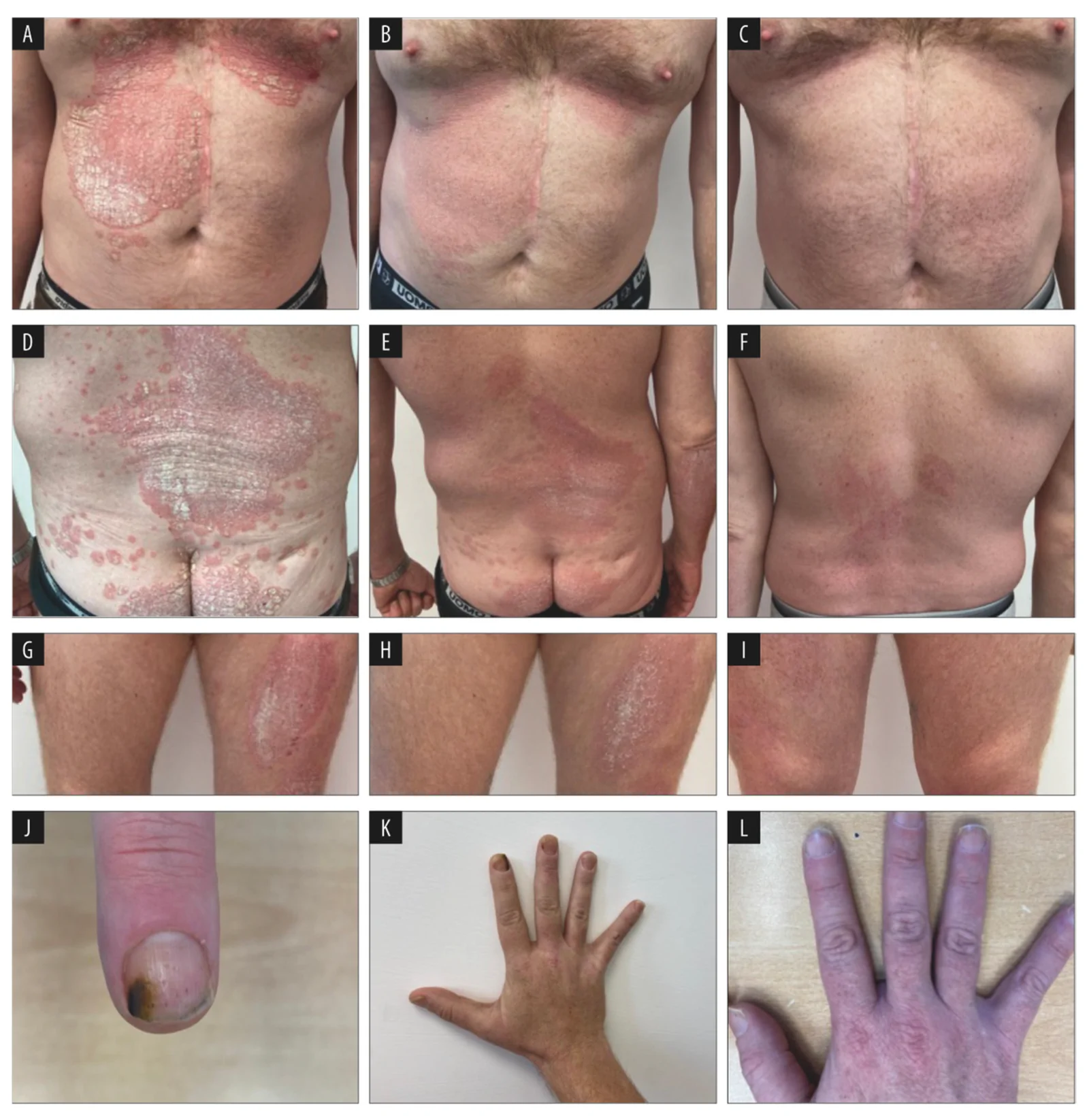
Case 4. (**A**,**D**,**G**,**J**). Week 0: extensive baseline erythematous and desquamative plaques involving the trunk (**A**), back (**D**), legs (**G**), and a concurrent *Pseudomonas aeruginosa* infection of the nail of the right index finger (**J**) before treatment with tildrakizumab; (**B**,**E**,**H**,**K**). Week 4: improvement of psoriatic lesions (**C**,**F**,**I**,**L**). Week 16: complete resolution of psoriatic lesions.

At the initial visit, the patient presented with evidence of impaired self-care, reflecting a depressive state and self-neglect attributed to the severity of his psoriasis and the prolonged inability to find effective treatment. Clinically, he had severe plaque psoriasis characterised by thick, indurated, and hyperkeratotic plaques localised on the trunk, limbs, scalp, and genital area (Figure 4A,D,G). The lesions were particularly pruritic, with an itch numeric rating scale (NRS) score of 9. On the elbows and legs, the plaques showed visible excoriations and scratch marks caused by scratching of lesions. Disease severity was scored 28 on PASI and a 4 on the Physician Global Assessment (PGA) scale. Additionally, the patient exhibited psoriatic nail involvement affecting all fingernails, complicated by a concurrent *Pseudomonas aeruginosa* infection of the nail of the right index finger (Figure 4J). The condition significantly impacted his quality of life, as reflected by the DLQI score of 16. No other comorbidities were reported.

Due to the severity of the disease, which affected high-impact areas and consequently compromised the patient’s quality of life and, particularly, his social life, we opted for tildrakizumab, an anti-IL-23 biologic with proven efficacy and acting faster than anti-TNF-α treatment. Standard pre-biologic screening protocols were executed and cleared prior to treatment. The treatment with tildrakizumab was initiated at a dose of 200 mg subcutaneously at Weeks 0, 4, and every 12 weeks. A risk-benefit analysis of concurrent biologic and antibiotic to treat nail infection was performed. Given the severity of the patient’s psoriasis with concurrent depression and the fact that nail infection was a minor infection treatable with a relatively short course of antibiotics, the decision was made to treat the patient simultaneously with tildrakizumab and levofloxacin at a dose of 500 mg twice daily for 10 days to treat the nail infection.

A rapid clinical response was observed. After 4 weeks, i.e., after a single 200 mg dose of tildrakizumab, the hyperkeratotic component resolved completely, with a marked reduction in skin infiltration; only erythema persisted (Figure 4B,E,H,K). At this time, the PASI score decreased to 6.3, the DLQI improved to 6, and there was a notable reduction in itch (NRS reduced to 2). By Week 16, complete skin clearance (PASI 0) was achieved, and the psoriatic nail involvement showed significant improvement, with the nail disease PGA score improving from 3 to 1, accompanied by the regrowth of healthy nail of the finger previously affected by the infection (Figure 4C,F,I,L). At the 24-week follow-up, the nail psoriasis achieved complete remission. The patient is still receiving tildrakizumab at 200 mg after 2 years of therapy with excellent clinical response and no adverse events.

## 3. Discussion

This case series illustrates the effectiveness of tildrakizumab across complex clinical scenarios, including extremely severe psoriasis with cardiovascular comorbidities, obesity, metabolic syndrome, prior biologic failure, difficult-to-treat areas, treatment interruption, and bio-naïve disease. In these patients, tildrakizumab was selected because of the possibility of tailoring dosing in patients with elevated body mass or high burden of disease. IL-23p19 inhibition provided rapid and sustained clinical improvement, supporting its use in both refractory and selected first-line settings.

Cardiovascular comorbidities are a common occurrence in psoriasis [13]. There is also a strong association between psoriasis and obesity and psoriasis and metabolic syndrome; obesity in psoriasis decreases efficacy of both conventional and biological therapies [14,15,16], whereas the involvement of hands has been linked to poor patient satisfaction and increased functional impairment [17]. Several studies have demonstrated the long-term efficacy and safety of tildrakizumab at the dose of 200 mg in obese patients and those with metabolic comorbidities [5,10,14,18,19]. There is also evidence for sustained long-term effectiveness of tildrakizumab in difficult-to-treat areas [11,20].

Being bio-naïve and having a short disease duration (up to 2 years) are clinical characteristics that help predict a good response to tildrakizumab. Also, early clinical improvement is an indicator of a lasting response [21,22]. However, IL-23 inhibition is also a good option for patients with failures of previous biologics, as illustrated by Cases 1–3 presented here. Compared to TNF-α inhibition, anti-IL23 agents offer a selective inhibition of the pathway involved in the pathogenesis of psoriasis versus broad inflammatory network inhibition [23]. The efficacy of anti-IL-17 and anti-IL-23 inhibition after adalimumab was previously shown [24]. Moreover, IL-23 is the master regulator of the inflammatory cascade in psoriasis, acting upstream of the IL-17 signalling, which means that patients who lost responsiveness to IL-17 inhibition respond to anti-IL-23 treatment [25,26]. Compared to the IL12/23 inhibitors, selective targeting of the p19 subunit, resulting in specific IL-23 inhibition, provided by tildrakizumab effectively suppresses disease-driving inflammation without impairing IL-12-mediated host defence [27]. IL-23 inhibition is also preferred due to superior safety [28].

Regarding safety, no adverse events occurred in the four cases, despite multiple comorbidities, including Case 2 for whom the longest follow-up is available. We wish to emphasise the importance of selecting treatments with favourable safety profiles and monitoring for potential adverse effects throughout therapy in patients with cardiovascular/metabolic comorbidities, including prior myocardial infarction and hypertension (Case 1), and obesity (Case 2 and Case 3) with metabolic syndrome (Case 2). Importantly, all patients described here reported an improvement in the quality of life, which is consistent with previous reports [29].

Admittedly, the number of patients included in this case series is small, which is a limitation of this report. Notwithstanding this, our case series adds to the growing real-world evidence supporting the use of targeted therapies such as tildrakizumab as an effective option in high-burden disease resulting in long-term remission duration in patients with psoriasis despite comorbidities, prior biologic failures, and involvement of high-impact areas.

## 4. Conclusions

Tildrakizumab represents an effective therapeutic strategy with a favourable tolerability profile for patients with psoriasis and clinical complexities such as comorbidities and the involvement of high-impact body areas. The patients described here are representative of this patient category. Therapeutic success promotes therapeutic alliance between doctor and patient.

## Figures and Tables

**Table 1:** Patient characteristics before starting treatment with tildrakizumab and treatment results.

	Case 1	Case 2	Case 3	Case 4
Age (years)	60	46	48	49
BMI	30.10	38	33.95	25.35
Disease duration (years)	20	15	20	26
Previous biologics	adalimumab secukinumab	infliximab adalimumab ustekinumab secukinumab	adalimumab tildrakizumab	None
Comorbidities	Cardiovascular disease, dyslipidaemia, hypertension	Obesity, metabolic syndrome	Obesity, uveitis, hepatitis C, depression	Nail infection, depression
Smoking	No	No	Yes	No
Duration of treatment with tildrakizumab (years)	1	4	1 + 2 *	2
Last follow-up (weeks)	54	216	108	108
BSA				
Week 0	93%	90%	80%	38%
Week 4	10%	15%	5%	31%
Last follow-up	0%	0%	0%	0%
PASI				
Week 0	57	24	57.6	28
Week 4	18	12	11.5	6.3
Last follow-up	0	0	0	0
DLQI				
Week 0	19	18	30	16
Week 4	5	6	0	6
Last follow-up	0	0	0	0

BMI, body mass index; BSA, body surface area; DLQI, dermatology life quality index; PASI, psoriasis area severity index. * Patient treated with tildrakizumab for a year who interrupted the treatment and was restarted on tildrakizumab a year later.

## Data Availability

All data generated or analysed during this study are included in this article. Further enquiries can be directed to the corresponding author.

## Abbreviations

The following abbreviations are used in this manuscript:

BMI	Body mass index
BSA	Body surface area
DLQI	Dermatology life quality index
HCV	Hepatitis C virus
IL	Interleukin
NB-UVB	Narrow-band ultraviolet B
NRS	Numeric rating scale
PASI	Psoriasis area severity index
PGA	Physician’s global assessment
PSSI	Psoriasis scalp severity index
TNF-α	Tumour necrosis factor-alpha
